# Monthly Summary

**Published:** 1891-09

**Authors:** 


### Monthly Summary.

Covering Gold Crowns with Glass —I would like to present to the society this evening a method for enameling the faces of all gold crowns. We all know how conspicuous gold crowns are in the mouth, which characteristic has always been very objectionable to me, and I have tried various devices for remedying that difficulty. Since the introduction of glass fillings, I have hit on a plan which removes almost entirely the unsightly appearance, and I thought it might be interesting to describe how I did it. After fitting the ordinary gold crown to the root, I remove the crown and fill it with modeling composition, to keep it from bending. Then, with the ordinary jeweler's saw, I cut out the lower part of the buccal surface of the gold crown, commencing at the cutting edge, leaving a ring of gold at the top. I then remove the composition and fit a piece of either thin platina or gold—I prefer the platina—to the inside of the crown, just to the edges of the opening made with the saw. I always thicken the crown at the cusps with solder, using twenty-carat solder, and, holding it over the burner, I solder the little piece of platina at one end, and then add the glass filling of the proper shade. The color depends much on how it is fused; if it is fused too much, it bleaches; if fused too little, the color is not brought out. After wetting the glass with water, I pack it in over the platina, and with some bibulous paper make it as smooth as possible; then I hold it over the alcohol lamp with the pliers. If not plump enough, I repeat the process till I have it contoured to my satisfaction. I have had some of these enameled crowns wearing in the mouth for six months, and there has been no change.

I have tried another way too,—that of grinding very thin pieces of porcelain from English teeth, or even American teeth, fitting them towards the surface, from the inside, and fusing glass around them. That gives the color a little better, but it is more work. Enameling by the first method can be done in ten minutes.—*Dr. J. B. Littig in N. Y. Odont. So., Inter.*

---

A GOLD CLASP, PERFECT IN FIT, EASILY MADE, AND NOT WEARING TO THE TOOTH CLASPED.—Take spring clasp-metal of proper width; bend into a hoop large enough to surround two-thirds of the tooth without touching it, attach to plate; drill three holes at carefully selected points; insert set-screws; spring over the tooth; tighten up your screws, and you will have a clasp, strong, tight, stable, which will closely fit an ill-shaped tooth without its having to be cut. The principle is that used in the surveyors tripod, which enables it to stand steady upon any irregular surface by the bringing of the three feet into the same plane.—*Dr. W. C. Wardlaw, in Headlight.*

---

OBSERVATIONS ON THE DECAY OF THE TEETH OF YOUNG MOTHERS.—1. The decay of the teeth shows a violation of the law of our beings. There is harmony and sympathy throughout our organisms. Pregnancy is a normal condition and is not responsible for decay of the dental organs.

2. Mothers who neglect the proper care of their mouths and teeth have decayed teeth, and that mothers' teeth decay in proportion to their density and the care bestowed on them.

3. That the sickness incident to childbirth and the care of small children takes so much time and care of the mother that she neglects her teeth for days, weeks and sometimes for months.

4. That the mother's maiden sisters and bachelor brothers have decayed teeth also.

5. The whole subject is summed up in the following, conditions being equal: Teeth decay—1st. In proportion to their density. 3nd. In proportion to their care and proper cleansing. Therefore, every mother and her maiden sisters should not only care for their teeth the best they know how, but they should visit a competent dentist frequently to have their mouths examined and get some wholesome instruction regarding the care of these most valuable organs.—*Archives.*

---

THE UNITED STATES SUPREME COURT DECISION IN "TOOTH CROWNS."—This decision was rendered April 27 in the case of Dr. Edward S. Gaylord, of New Haven, Conn., and in opposition to the claims of the Tooth Crown Company.

It will be remembered that in 1887 a suit was brought against Dr. Gaylord in the United States Circuit Court in New Haven. The judges decided the patent invalid. The case was then appealed to the Supreme Court. It reached an argument some weeks ago, the justices finally reaffirming the decision of the Circuit Court.

This conclusion will be regarded with satisfaction by the profession. Much is due to Dr. Gaylord for his long and persistent fight, and it would seem proper that this gteat service should receive recognition in a form worthy the man and the work.—*International Dental Journal.*

